# Seminal bacterial contaminations: Probable factor in unexplained recurrent pregnancy loss 

**Published:** 2013-11

**Authors:** Ali Nabi, Mohammad Ali Khalili, Iman Halvaei, Jalal Ghasemzadeh, Ehsan Zare

**Affiliations:** *Research and Clinical Center for Infertility, Shahid Sadoughi University of Medical Sciences, Yazd, Iran*

**Keywords:** *Recurrent pregnancy loss*, *Bacteria*, *Semen*

## Abstract

**Background:** It is estimated that about 50% of causes of recurrent pregnancy loss (RPL) cases remain unknown. Sperm factors are suggested to have probable role in cases with RPL.

**Objective: **The goal was to determine the possible relationship between semen bacterial contaminations with unexplained RPL. Also, the correlation between number of bacterial colony and sperm chromatin condensation was examined.

**Materials and Methods:** This study consisted of 30 fertile men (group A) and 30 infertile (group B) men with unknown RPL. Semen collection and analysis were done according to WHO manuals. Sperm count and motility were evaluated by Makler chamber. Eosin-Nigrosin and Papanicolaou staining methods were applied for viability and morphology assessment, respectively. The semen samples from both groups were cultured for aerobic bacteria. Aniline blue (AB) and toluidine blue (TB) staining methods were applied for evaluating sperm chromatin condensation.

**Results: **The numbers of colonies were significantly higher in group B when compared to group A. Also, S. aureus and E. coli contaminations showed significant differences between two groups. Both AB+ and TB+ sperm cells showed significant increase in group B compared to group A. There was a significant negative correlation between colony number and progressive motility (p=0.01), and sperm viability (p=0.007). In addition, positive correlations were found between colony number and AB^+^ (p=0.001) and TB+ (p=0.004) as well.

**Conclusion:** Bacterial contaminations in semen of men from RPL couples had significantly higher levels when compared to fertile controls. Presence of microorganisms in semen may be correlated with irregular sperm parameters and quality.

## Introduction

Infection in the urogenital tract is one of the causes which can lead to male infertility. It is estimated that about 15% of infertile couples suffer from male urogenital tract infection (1). It appears the main mechanism induced by different microorganisms to cause infertility is not fully understood because diagnosis of many infections maybe difficult due to lack of any symptoms during infection. Numerous microorganisms including bacteria and viruses can lead to infection of genital system in men. Chlamydia trachomatis (C. trachomatis) is considered as the most common organism involved in acute non-bacterial prostatitis and urethritis also can infect epididymis, vas deferens and testis, as well (2). 

Recurrent pregnancy loss (RPL) is the abortion of at least two consecutive pregnancies in the first or early second trimester of gestation (3). RPL may have different etiologies like anatomical, genetic, psychological, thrombotic and immunological defects. It is estimated that about 50% of RPL cases remain unknown, maybe due to different factors involved in RPL (4). Sperm factors are suggested to have probable role in RPL cases (5, 6). Many researchers tried to pinpoint the probable causes of sperm factors related to RPL, such as conventional sperm parameters (e.g. concentration, motility, morphology, viability) as well as acrosomal status, presence of leukocytes in seminal plasma, lipid peroxidation of sperm plasma membranes, antioxidant capacity of seminal plasma, and sperm chromatin integrity (6-10). 

Ombelet *et al* designed prospective study in order to evaluate sperm parameters between fertile and infertile patients (11). They found that the rate of bacteria in seminal fluid in infertile men could be similar to fertile, and it seems that the clinical significance of presence of microorganisms in semen is still matter of debate. Talebi *et al *evaluated sperm chromatin packaging and DNA integrity in couples with unexplained recurrent spontaneous abortion (12). They stated that sperm cells from recurrent abortion have less chromatin condensation compared to fertile group. Also DNA integrity had better quality in fertile group compared to recurrent abortion group. There are several techniques in order to assess sperm DNA integrity (13-14). Aniline blue (AB) and toluidine blue (TB) staining methods are cytochemical assays which can detect sperm chromatin condensation anomalies. 

We hypothesized that the bacterial contamination may have negative impact on sperm function and quality. To our knowledge, there are rare studies about the probable correlation of bacterial contamination and RPL. The main goal was to determine the possible relationship between the results from semen bacterial contaminations and unexplained RPL. Also, we compared the sperm chromatin packaging (using AB and TB tests) in RPL cases with fertile controls. Finally, the correlation between detected colony number and progressive motility, normal morphology, viability and DNA packaging tests were addressed.

## Materials and methods

**Patients**

This case control study involved 60 individuals which were divided into two groups of A (fertile men, n=30) and B (infertile men with RPL, n=30). This study was done from November 2012 to May 2013. Inclusion criteria for groups A and B were to have a child within the last two years and to have at least two recurrent miscarriages in last two years, respectively. Exclusion criteria were smoking and variciselectomy. The semen samples from fertile group were obtained from men who referred to Akbari Clinic, Yazd, Iran for vasectomy. 

The specimens were entered into study chronologically. Comprehensive examinations for group B were done and it was showed that all criteria are normal and the patients were considered as idiopathic cases. Individuals in both groups had no clinical signs, neither symptom of infections in their lower genital tract. The semen samples (group B) were obtained from patients who were referred to Yazd Research and Clinical Center for Infertility. All men were Muslim and had circumcised during their childhood. All the patients were asked to sign the consent forms. This study was approved by our institute ethic committee.

**Semen collection and analysis**

**Figure 1:**
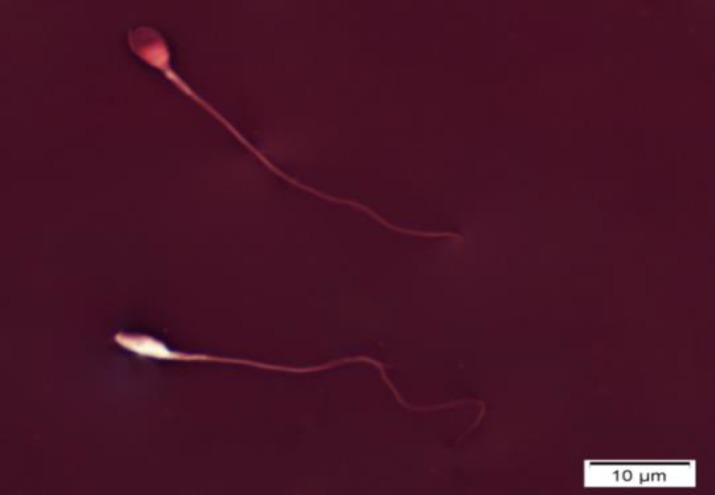
Evaluation of human sperm viability using eosin-nigrosin staining. a: unstained (white) is alive spermatozoon, b: stained (red) spermatozoon is dead

**Figure 2:**
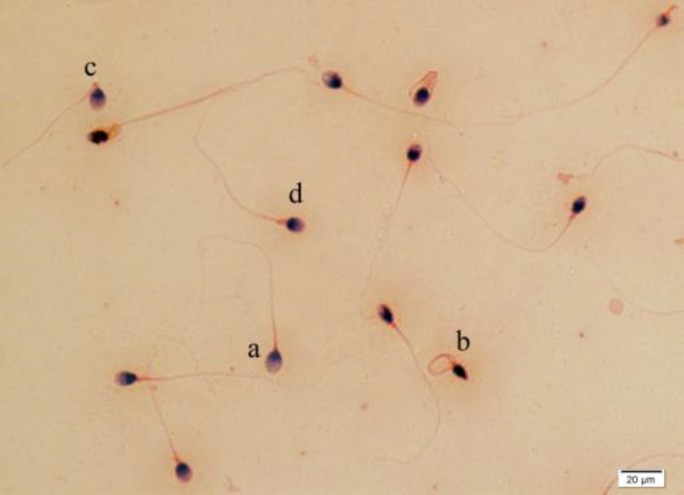
Papanicolaou staining for sperm morphological evaluation. a: normal sperm cell, b: coiled neck sperm cell, c: bent neck sperm cell, d: thick neck sperm cell

Ejaculates were collected by masturbation in a wide-mouth sterile container. Abstinence period was considered 3-7 days. Semen analysis was done according to WHO (2010) manuals (15). Sperm count and motility were evaluated by Makler chamber as described previously (16-17). Eosin-Nigrosin staining test and Papanicolaou staining were applied for viability and morphology assessment, respectively (Figure 1 and 2).

**Semen culture**

For assessment of bacterial infections, 10µl of semen sample was divided into two culture media: blood agar and eosin methylen blue (EMB) for detection of gram positive and gram negative microorganism, respectively. After 24 h of incubation at 37^o^C, the presence of microbial and bacterial colonies were evaluated. After gram staining, differential tests were used for detection of microorganism species. Catalse test was applied for gram positive cocci in order to determine streptococcus and staphylucus. Gram positive cucci, with both coagulase and manitol fermentation tests positive, were considered as *S. aureus*. The number of colonies were counted using colony counter. 

**Aniline blue (AB) staining**

**Figure 3:**
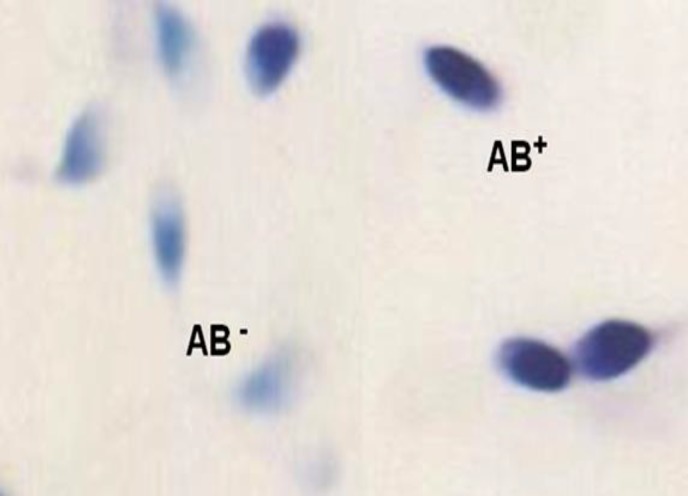
Aniline blue staining method. For sperm morphological evaluation. Light blue sperm heads show normal DNA and dark blue sperm heads show sperm DNA damage

Aniline blue (AB) staining, as a cytochemical staining, stains lysine-rich histones and is considered as a standard test for detection of defects in condensation of sperm DNA chromatin (18). The air-dried smears from fresh semen were fixed in 3% buffered glutaraldehyde in 0.2 M phosphate buffer (pH=7.2) at room temperature. After staining of each smear with AB stain (in 4% acetic acid, pH=3.5), the slides were checked for presence of normal and abnormal spermatozoa using light microcopy (Olympus Inc., Tokyo, Japan). Unstained or pale blue stained cells and dark blue cells were considered as normal (AB-) and abnormal spermatozoa (AB+), respectively. At least 200 sperm cells were evaluated in each slide and the normal and abnormal spermatozoa were reported as percentage (12) (Figure 3).

**Toluidine blue (TB) staining**

**Figure 4:**
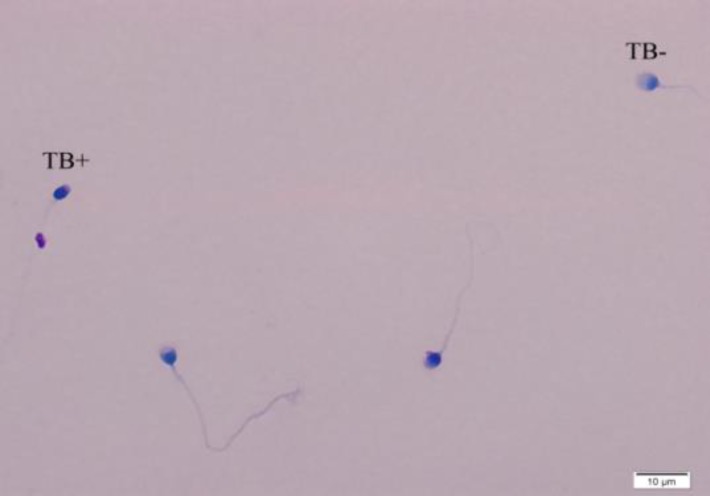
Toluidine blue staining method for sperm morphological evaluation. Light blue sperm heads show normal DNA and dark blue sperm heads show damage to DNAA

TB stain can bind to phosphate groups of DNA strands and reveal the chromatin condensation status (19). Briefly, the air-dried smears from fresh semen were fixed in 96% ethanol-acetone (1:1) at 4^o^C. The slides were then put in 0.1 NHCl at 4^o^C for hydrolysation. After rinsing with distilled water, the slides were stained with 0.05% TB for 10 min. Pale blue sperm cells were considered as normal and dark blue or violet or purple spermatozoa were categorized to abnormal cells. At least 200 spermatozoa were checked for each slide and the normal (TB-) and abnormal (TB+) sperm cells were reported as percentage (20) (Figure 4).

**Statistical analysis**

The data were shown as mean±SD. Independent samples T test and Mann-Whitney U test were used as parametric and non-parametric tests, respectively, in order to compare the quantitative data between groups. Fisher’s exact test was applied for comparison of qualitative data between two groups. Spearman test was used in order to find out the correlation between detected colony number and progressive motility, normal morphology, viability and DNA packaging tests (AB and TB). All hypotheses were considered two tails and significant level was set at 0.05.

## Results

Of total of 35 and 47 semen samples in group A and B, 30 and 30 specimens were met inclusion criteria, respectively. There were no significant differences for men age, semen volume and sperm concentration between two groups. But progressive motility, normal morphology as well as sperm viability was statistically higher in group A compared to group B. Regarding sperm DNA integrity status, both AB+ and TB+ sperm cells showed significant increase in group B compare to group A (Table I). Table II shows semen culture outcomes between two groups. A total of 3 and 6 different types of bacterial species were detected in groups A and B, respectively. Only 3 samples in group B showed negative culture while no bacteria were grown after semen culture in 22 samples in group A. 

The most prevalent bacteria in recurrent abortion and control groups were *S. aureus* and S. *epidermidis*, respectively. The numbers of colonies were significantly higher in group B when compared to group A (117666.67± 90551.31 vs. 28666.67±58768±0.06). In addition, *S. aureus *and *E. coli *in groups A and B were 0 vs. 9/30, and 0 vs. 6/30, respectively. There was a significant negative correlation between colony numbers and progressive motility (p=0.01, correlation coefficient=-0.31), and sperm viability (p=0.007, correlation coefficient=-0.34). Whereas, positive correlations were found between colony number and AB (p=0.001, correlation coefficient=0.41) and TB (p=0.004, correlation coefficient=0.37) as well.

**Table I:** Comparison of different parameters between two groups of recurrent abortion and control

**Parameters**	**Control** **(group A)**	**Recurrent abortion** **(group B)**	**p-value**
Male age	31.43 ± 7.00	31.97 ± 4.45	0.16*
Semen volume (ml)	3.21 ± 1.37	3.23 ± 1.25	0.95^#^
Sperm count (×10^6^/ml)	109.73 ± 53.05	95.33 ± 54.62	0.22*
Round cell (×10^6^/ml)	0.53 ± 0.81	1.03 ± 1.73	0.58*
Progressive motility (%)	64.27 ± 6.4	51.3 ± 12.36	<0.0001^#^
Normal morphology (%)	40.33 ± 9.92	25.53 ± 15.37	<0.0001*
Viability (%)	85.43 ± 4.77	70.2 ± 12.81	<0.0001^#^
TB+ (%)	34.3 ± 8.3	61.23 ± 20.5	<0.0001*
AB+ (%)	36.93 ± 10.16	65.67 ± 21.55	<0.0001*

The data were presented as mean±SD.

TB: Toluidine blue

AB: Aniline blue

* : Statistical analysis using Mann-Whitney U test

# : Statistical analysis using independent samples T test

**Table II:** Comparison of prevalence of bacteria detected from two groups of recurrent abortion and control

**Parameters**	**Control** **(group A) (n=30)**	**Recurrent abortion** **(group B) (n=30)**	**p-value**	**OR (95% CI)**
Colony (CFU/ml)	2.8 x 10^4 ^± 58768 ± 0.06	117666.67 ± 90551.31	<0.0001*	
Negative culture	22	3	<0.0001^#^	0.04 (0.009-0.1)
*S. aureus*	0	9	0.001^#^	26.9 (1.4-488.6)
*S. saprophyticus*	3	4	1^#^	1.3 (0.2-6.7)
*S. epidermis*	4	5	1^#^	1.3 (0.3-5.4)
*E. coli*	0	6	0.02^#^	16.1 (0.8-301.8)
Streptococci beta hemolytic	0	2	0.4^#^	5.3 (0.2-116.4)
Pseudomonas Sp.	0	1	1^#^	3.1 (0.1-79.2)
Enterococcus Sp.	1	0	1^#^	0.3 (0.01-8.2)

OR: odds ratio

CI: confidence interval

CFU: colony-forming unit

* : Statistical analysis using Mann-Whitney U test

# : Statistical analysis using Fisher’s exact test

## Discussion

Unknown recurrent abortion could have different etiologies. In this study, it was shown that bacterial infection in semen might be related to this phenomenon in infertile couples. Our results showed that some bacterial species were significantly higher in recurrent abortion group compared to fertile controls. We found that sperm progressive motility, viability and normal morphology were significantly higher in group A compared to group B, while sperm count and semen volume were the same between groups. The relationship between conventional sperm parameters and recurrent abortion is matter of debate in literature (12, 21). 

It was shown that microorganisms in semen can impair sperm function via agglutination of motile spermatozoa, impairment in acrosome reaction, morphology, and induction of apoptosis (22, 23). Some bacteria may affect sperm function via their pili. Gram positive bacteria, like enterococcus, can bind to mannose receptor in sperm surface and induce sperm damage (23, 24). It is shown that there is an inverse correlation between presence of some bacteria in semen with sperm count and motility (25). 

In vitro studies using scanning electron microscopy and light microscopy have shown that bacteria (i.e. *E. coli *and mycoplasmas) may lead to alteration in sperm morphology (26, 27). It was stated that bacterial infection in semen of infertile men can affect the semen quality (28). Prabha *et al* have demonstrated an immobilizing factor production by *E. coli*. They showed that incubation of sperm with *E. coli* can impair sperm motility and morphology (26). 

In our study, *E. coli* was detected in 20% of specimens in group B which is similar to previous report by our laboratory (29). Previously we found that *E. coli* in 22.2% of infertile samples resulted in significant reduction in progressive motility, normal morphology as well as sperm viability. It has also been proposed that the reduction of sperm motility by leukocytes and *E. coli* could be due to depletion of adenosine triphosphate (30). It has been reported that in vitro incubation of sperm with *E. fecalis*, *E. coli* and *S. aureus* may induce sperm apoptosis. This could be due to adhesion of bacterial pilus or flagellum to the sperm, resulting in activation of caspases followed by DNA damage (23). 

In addition, bacteria toxin has been indicated as another mechanism for sperm apoptosis. Round cells are routinely checked at andrology labs, which are representatives of germ cells or leukocytes (15). It was stated that presence of leukocytes in semen can impair semen quality and sperm function (31). Our data showed that the rate of leukocytes had increasing trend in group B. The presence of leukocytes in semen could be a source of producing reactive oxygen species (ROS). It is well known that ROS can induce sperm membrane and DNA damage. 

Our data showed that round cells, as a representative of leukocytes, had increasing trend in group B which was also more than WHO cut-off point of ×10^6^/ml. Presence of leukocytes in semen could be subsequently due to presence of microorganisms in semen. Therefore, bacteria in semen can indirectly induce ROS production and finally impair sperm viability and DNA integrity before fertilization and embryo development in later stages. TB can bind to phosphate groups of DNA strands and shows the rate of sperm nuclear chromatin condensation. Our results were in line with others that TB+ spermatozoa could be detected higher in recurrent abortion cases compared to fertile controls (12). 

This could verify paternal role, especially the role of sperm DNA in etiology of abortion. Presence of microorganisms in semen may alter sperm DNA status directly or indirectly. It was shown that sperm chromatin condensation and DNA integrity status can affect fertility potential. When the sperm chromatin is less condenses, the susceptibility of sperm to environmental factors is more. AB staining test shows extra lysine-rich histone proteins. Our results showed that the rate of AB+ spermatozoa was significantly higher in group B compared to A which was in agreement with others (12, 21). In agreement to our results, Talebi *et al* found that AB+ spermatozoa had significant increas in unexplained recurrent spontaneous abortion group compared to controls (12). 

The data generated from this study showed that the rate of TB+ sperm cells had significant increase in group B. Our data showed that about 61% of spermatozoa were TB-reacted in group B. This rate was 82% in Talebi *et al* study (12). While, Rybar *et al* did not find chromatin impairment (using SCSA) in semen samples contaminated by chlamydia, ureaplasma and mycoplasma strains compared to non-contaminated semen samples (32). Gallegos *et al* demonstrated that sperm DNA fragmentation (using SCD) will be increased in contamination of semen with mycoplasma and Chlamydia (33). 

Since different strategies have been used for evaluation of integrity of DNA, it has become difficult to compare these studies. In this study, we have tried to detect only aerobic microorganisms. On the other hand, the clinical significance of the presence of anaerobic microorganisms in semen is still controversial issue. This has further exacerbated due the difficulties associated with the anaerobic culturing of semen. Probable role of anaerobic bacteria on semen quality and fertility potential needs to be further elucidated. 

Also Chlamydia, as intrinsic microorganism, needs to be clarified by HELLA test which was not feasible to do in this study and is suggested to evaluate in future investigations.

## Conclusion

This study showed that number of positive bacterial culture and colony number were significantly higher in semen of men from infertile couples due to recurrent abortion compared to fertile controls. In addition, it was shown that *S. aureus* and *E. coli *had significant increase in RPL cases. It could be suggested that comprehensive bacteriological examinations with subsequent antibiotic sensitivity assay and antibiotic therapy would be necessary before admission of infertile men into infertility treatment, especially in RPL cases.
